# Fourier transform infrared spectroscopic technique for analysis of inorganic materials: a review

**DOI:** 10.1039/d5na00522a

**Published:** 2025-08-26

**Authors:** Kazi Al-Amin, Md. Kawsar, Md. Tariqur Rahaman Bhuiyan Mamun, Md. Sahadat Hossain

**Affiliations:** a Department of Applied Chemistry and Chemical Engineering, Noakhali Science and Technology University Noakhali Bangladesh kawsar.acce@gmail.com; b Glass Research Division, Institute of Glass & Ceramic Research and Testing, Bangladesh Council of Scientific and Industrial Research (BCSIR) Dhaka 1205 Bangladesh saz8455@gmail.com

## Abstract

FTIR is a very important analytical technique that is widely used for the detection and analysis of inorganic materials. It has a wide range of applications, from chemical composition analysis, structure identification, and phase identification to surface analysis of inorganic materials. Despite its broad effectiveness in the arena of material science, a detailed review of inorganic materials is scarce. Most existing research has focused mainly on the synthesis of organic molecules or specific types of inorganic materials like ceramics and minerals. Also, a few studies discuss how advancements in FIIR technology can be employed to more precisely analyze these materials. Furthermore, there is a lack of knowledge about how FTIR complements other methodologies like X-ray diffraction (XRD) and Raman spectroscopy, especially for inorganic materials. This research tries to bridge these gaps by a comprehensive review of the topic of FTIR's role in inorganic material analysis. This paper will also cover theoretical background, practical application in chemical composition structure identification, phase identification, recent advancements, and resolution data acquisition and analysis. Furthermore, this article will discuss how FTIR compares with other analytical methods by highlighting its pros and cons. This review will serve as an excellent guide for researchers and industrial professionals to use FTIR spectroscopy more effectively in their work on inorganic materials analysis.

## Abstract 2

FTIR is a very important analytical technique that is widely used for the detection and analysis of inorganic materials.

## Graphical abstract

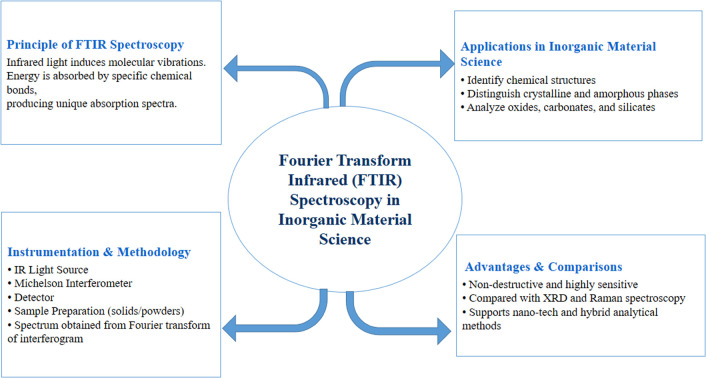

## 1 Introduction

FTIR is performed to help not only scientists but also engineers understand the molecular nature and structure of the material within the context. The underlying idea is that substances have specific absorption bands in the infrared range, which induce various vibrations in the chemical bonds. Chemicals bonded in molecules vibrate, and these vibrations are unique according to different functional groups attached to the molecules. When plotted on a graph, they form a spectrum that is considered to be the molecular signature of any material type.^1,2^ In the field of materials science, it is often necessary to analyze the molecular composition and structure of a given material for various needs, such as creating a new material or verifying the correctness of the content during the manufacturing process. FTIR is essential in this area as it can furnish more information about the molecules of a large variety of materials, which can be solid, liquid, or gas. In addition to the properties mentioned above, in material science, FTIR can also be used to identify the functional groups present in a molecule, thereby enabling scientists to determine the molecular structure of a material. This is even more critical when investigating polymers, ceramics, composites, and other advanced materials whose performance and properties are governed by their chemical structure.^1,3^ When it comes to analyzing materials like oxides and carbonates commonly present in minerals and ceramics, as well as glasses, using methods such as FTIR spectroscopy becomes highly valuable for understanding their properties and composition better by looking at their specific vibrational patterns and structures, which can reveal insights into their purity and crystallinity levels effectively. For example, studying minerals using FTIR spectroscopy can help distinguish between different silicate formations, such as chain silicates or sheet silicates, by examining their distinctive spectral characteristics closely.^4^ Proof of its effectiveness in the field is that Fourier Transform Infrared (FTIR) spectroscopy has become an indispensable tool for the detection and analysis of inorganic materials with a wide range of applications from chemical structure identification to phase transformation studies.^5^ Among these techniques, FTIR has been extensively used in the arena of material science, but a detailed review describing it as an apt tool for the analysis of inorganic materials is scarce. Current literature has focused on the synthesis of organic molecules or specific types of inorganic materials (*e.g.*, ceramics and minerals), with little discussion on the breadth of applicability to a wide class of inorganic systems.^6,7^ Furthermore, FTIR instrumentation and methodologies have advanced in a technologically driven way, but few studies probe how those enrichments may influence inorganic material analysis. This presents a void in knowledge about how advancements in FTIR technology can be employed for more precise analysis of these materials.^8^ Furthermore, we lack knowledge on how FTIR complements other methodologies (alongside X-ray diffraction (XRD) and Raman spectroscopy, for example), especially for inorganic materials. Although individual studies such as these have compared these methods, a more in-depth comparison is required to illustrate not only that the two are complementary, but also elucidate under which scenarios one method may be preferred over the other.^9^ In these gaps, we try to bridge them by a comprehensive review of the topic of FTIR spectroscopy in inorganic material analysis. The paper will provide an outline of the theoretical background and practical utility of FTIR, highlighting examples from its application to chemical composition, phase identification, and surface properties.^10^ Advances in abilities such as resolution, data acquisition, and handling of recent FT-IR technology that have relevance to the inorganic materials community will also be discussed.^11^ Additionally, this article will discuss how FTIR compares with other analytical methods by highlighting its pros and cons. It will also serve as an excellent guide for researchers and industrial practitioners to use FTIR spectroscopy more selectively in their work on inorganic materials. Overall, the review serves to address a current lack of understanding regarding FTIR for inorganic material analysis and provides insight into the potential it promises in future applications.^3,10^

## 2 Principles of FTIR spectroscopy

### 2.1 Basic theory behind FTIR

At the most basic level, Fourier Transform Infrared Spectroscopy (FTIR) relies on the fact that different bonds in a molecule will vibrate at very specific frequencies when exposed to infrared (IR) light. Such vibrations are directly related to the molecular structure, rendering FTIR a very important tool for the identification and characterization of chemical compounds. Fundamentals of FTIR: the backbone of FTIR is the molecular vibrations. A chemical bond can be viewed as a spring linking the atoms in any molecule. The energy from the infrared light can be absorbed by a molecule, which makes these bonds vibrate in different modes. Such vibrations may be stretching (in which the separation between atoms changes), bending (where the angle between bonds changes), or a more complicated movement of multiple atoms. These vibrations have their own frequencies, which will differ according to the masses of the atoms being vibrated, and how strongly each atom is bonded to its neighbours. The higher the frequency of vibration, the lighter the atoms and/or the stronger the bond (the heavier, weaker). Since different types of chemical bonds and functional groups within a molecule have unique vibrational frequencies, each molecule has a unique infrared absorption pattern. This spectrum acts like a molecular fingerprint that can be utilized to identify and analyse the substance.^12,13^ FTIR functions by introducing a pattern to an enormous scope of infrared light and measuring how much light is absorbed at each frequency. The output is an infrared absorption spectrum showing the absorbance (sometimes transmittance) of the sample *versus* frequency (or wavelength). The peaks in the spectrum correspond to particular vibrational modes of the molecule, and, with some analysis, its molecular structure and composition can be deduced from it. The Fourier transform portion of FTIR is related to a mathematical data processing step necessary to convert the raw signal recorded by the spectrometer into an actual spectrum. FTIR is different from previous infrared spectroscopy methods, which measure one wavelength at a time; FTIR measures all of them together. When the light that has been transmitted through the sample is deformed, an interference pattern will be recorded in a time-domain form. Applying the Fourier transform converts this time-domain signal to a frequency-domain spectrum that is easier to interpret and analyse.^12,14,15^

### 2.2 How FTIR works: from infrared radiation to spectra

**Fig. 1:**
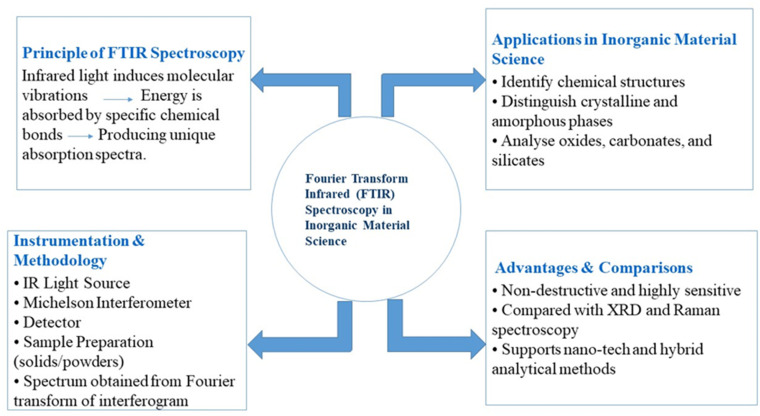
Graphical representation of the FTIR principle and instrumentation.

The FTIR spectroscopy itself is a multi-step process of generating infrared radiation, interacting between this stimulus and the sample, and physicochemical processing of the resulting data to form a spectrum. The working principle (Fig. 1) of FTIR, in a few steps, looks like this: emission of Fourier transform infrared radiation by the source inside the spectrometer. Usually, this is an electrically heated filament that emits a wide range of infrared light. This emits light ranging in wavelength from roughly 2.5 to 25 μm (4000–400 cm^−1^ wavenumbers, respectively).^13,16,17^ The infrared light is introduced into an interferometer, a key element of FTIR spectrometers. This beam splitter is part of the interferometer used, which splits incoming light into two beams. One beam goes to a mirror that is fixed in place, but another goes to a movable one. These two beams are then recombined by the beamsplitter, and since the movable mirror introduces a change in optical path length for one of them compared to the other, an interference pattern is created in the light that is recombined. This interference pattern, termed an interferogram, encodes information from not just one wavelength of light but from all wavelengths that have traversed the sample. The movable mirror is then continuously moved to the left and right of the neutral zero point, obtaining the interferogram based on coordinates as a function of the position of such a mirror.^13^ The infrared beam that has been recombined, and therefore contains the interference pattern information, is transmitted through or reflected from the sample. Based on their vibrational frequencies, specific wavelengths of light will be absorbed by the molecules as they interact with the sample. The remaining light interacts with the sample, or the rest of that light gets reflected and goes to the detector.^15,18^ The light travels on to a detector after passing through the sample. As the sample modulates the light, it is detected and recorded to measure the intensity across various wavelengths. What is actually detected, however, is still an interferogram-a complex signal that cannot be directly read out as an absorption spectrum.^15,16^ A mathematical operation (a Fourier transform) is used to convert the interferogram into something more interpretable. The interferogram contains the time-domain data, which is then mathematically transformed into frequency-domain data that displays the amount of absorption at each wavelength/frequency as a spectrum. The spectrum is normally displayed with the wavenumber (inverse of wavelength) on the *x*-axis and the absorbance (or sometimes transmittance) on the *y*-axis. Each peak in the spectrum is related to an absorption of infrared light by the sample and thus corresponds to specific molecular vibrations.^15,16^ The last step is to interpret the spectrum and determine the molecular structure of the sample. Different peaks in the FTIR spectrum correspond to different vibrational modes of the molecule. Through deconvolution of the location, strength, and shape of the peaks, it is possible to quantify bond types of a molecule and, in many cases >functional groups and molecular structure. A sudden peak around 1700 cm^−1^ usually indicates carbonyl functionality (C=O) in the molecule, for instance. Likewise, a broad peak in the range of 3200–3600 cm^−1^ can be related to hydroxyl (O–H) groups. It can then be used to accurately identify the material by comparison of the observed spectrum with reference spectra using software for spectral analysis. FTIR spectroscopy operates based on the principle of absorption of infrared light by a sample as a function of wavelength, resulting in an infrared spectrum, which is formed due to molecular vibrations within the sample. One finally studies this spectrum to determine the molecular composition, and in some cases, the molecular structure of a sample. It is the simultaneous measurement, mathematical transformation, and detailed analysis that make FTIR a powerful tool for molecular characterization of many types of materials.^15^

## 3 FTIR instrumentation and methodology

### 3.1 Components of FTIR spectrometers

FTIR spectrometers are sophisticated instruments that work by measuring the infrared absorption of materials to obtain information about their molecular structure. FTIR Spectrometer. The key components of an FTIR spectrometer consist of the Infrared Light Source, Interferometer, Sample Holder, Detector, and Computer Systems for Data Processing. These are all important components of the process used to generate a high-quality infrared spectrum.

The light source of an FTIR spectrometer is usually a broadband infrared emitter, such as a globar (a silicon carbide rod heated at high temperatures) or a nichrome wire. These sources emit an almost Continuum Infrared throughout a broad range of wavelengths, typically 4000 to 400 cm^−1^ (2.5 to 25 micrometers in wavelength). The stability and strength of the light source are critical in obtaining reproducible and precise spectra.^19^ The center of the FTIR spectrometer is the interferometer, which develops an interference pattern in this seamless infrared light that contains information about all wavelengths of light at once. The Michelson interferometer is the most frequently used interferometer in FTIR instruments. This creates an interference pattern or an interferogram, which is a map of the complex amplitude of many different frequency components of the light source.^13^ A sample holder is the place where the sample will be located for other analysis processes. Various holders or accessories are used based on the state of the sample (*e.g.*, solid, liquid, or gas).^15,20^ The infrared light passes through the sample and finally reaches a detector that measures the intensity of the emitted light. It turns the infrared light into an electric signal, and then this is sent to the computer for processing.^13,15^ A computer is essential to operate an FTIR spectrometer. It manages the translation of the movable mirror inside the interferometer, saves the acquired interferogram, and carries out a Fourier transform on the recorded interferogram to obtain a spectrum and features for data analysis and interpretation. Typically, modern FTIR systems come with advanced spectral analysis and bank compare features stacked in their software packets, along with an automated reporting solution.^13,20^

### 3.2 Sample preparation techniques for inorganic materials

FTIR spectra of metals and some insoluble salts can give highly reproducible results; however, they are often difficult to analyze, as most inorganic samples are heterogeneous in nature. Sample preparation techniques differ according to the physical state of the material and the kind of analysis being performed.

#### 3.2.1 Solid samples

##### 3.2.1.1 KBr pellet method

A common method used for solid inorganic sample preparation involves grinding the sample into a fine powder and subsequently mixing it with potassium bromide (KBr) powder. A hydraulic press compresses this mixture into a thin, transparent pellet. KBr has a crystalline structure, and it is transparent in the infrared region, thus making sample absorbance easy without any interference. It is especially applicable to crystalline materials.

##### 3.2.1.2 Mull method

This method involves the preparation of a mull of powdered sample with a viscous liquid (typically a mineral oil, Nujol). The mull is then sandwiched between two infrared transparent windows (KBr or NaCl), and the spectrum is recorded. It is commonly used for samples that do not give a stable pellet or react with KBr.

##### 3.2.1.3 ATR (attenuated total reflectance)

**Fig. 2:**
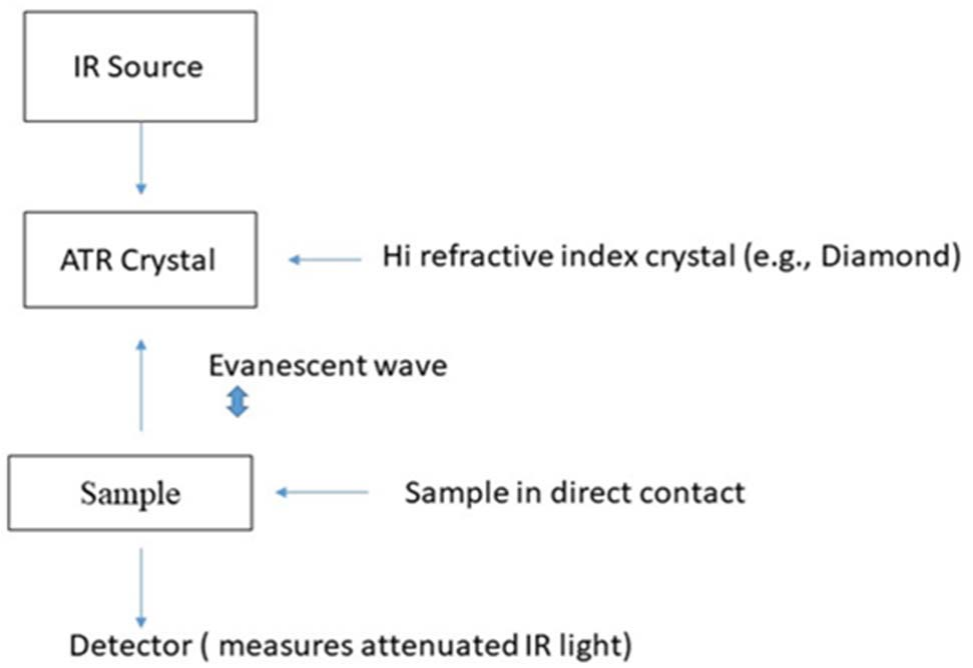
ATR graphical representation.

Ideal for many inorganic materials, especially those that are not easy to prepare as pellets, ATR is a common technique used (Fig. 2). In ATR, once a sample is in contact with the crystal (of high refractive index, such as diamond or zinc selenide). When infrared light is sent into the crystal, it is reflected at the interface between the crystal and sample the amplitude of the penetrating depth in the sample. Such a method is less time-consuming and is appropriate for powders, thin-films, and other solid materials.^19,20^

#### 3.2.2 Liquid samples

##### 3.2.2.1 Transmission cells

A small volume of the sample is placed between two infrared-transparent windows (KBr or CaF_2_), forming a transmission cell for inorganic liquids. The cell is positioned along the route of the infrared beam, and a spectrum is detected. In the case of aqueous solutions, it is necessary to utilize materials with a higher protection level against water damage, such as CaF_2_.

##### 3.2.2.2 ATR

ATR can also be performed on liquid samples; in the case of ATR, the liquid is just dropped on the ATR crystal, and the spectrum is collected. It is an ultra-fast process without any special preparation.^15,18^

#### 3.2.3 Gas samples

##### 3.2.3.1 Gas cells

Long enclosed tubes with windows at either end that are used for the analysis of gaseous inorganic substances. In this technique, a gas sample is put into the cell where it passes through infrared light, and its spectrum can be recorded. The gas cell is typically outfitted with mirrors so as to increase the effective path-length, thereby ensuring high sensor sensitivity for gases at low concentration levels.^19,20^

### 3.3 FTIR data acquisition and processing

FTIR spectra: plays a dual role as both an experimental technique for data acquisition (interferogram collection and Fourier transform) and analysis (spectrum interpretation). Finding an appropriate set of procedures to follow is important for any expected quality of results.

#### 3.3.1 Capturing the interferogram

The first step in this method is the acquisition of the interferogram, which is a type of raw data collected by FTIR spectrometers. It indicates the amount of light that is registered when the movable mirror in the interferometer moves. While the interferogram has all of the information necessary to yield the spectrum, it is not useful in this form. The quality of an interferogram is determined by the accuracy associated with the used interferometer, the stability of the light source used, and the sensitivity of the detector.^13^

#### 3.3.2 Performing the Fourier transform

Once we have the interferogram, the next step is to pass it through a mathematical process known as the Fourier transform, giving us its corresponding spectrum in our desired unit. This procedure gives the frequency spectrum of the absorbance (or transmission) *versus* wavenumber (the reciprocal of wavelength) by decomposing the complex signal into its complex Fourier components. The actual Fourier transform is a mathematical operation performed by the computer system and is usually automated in the spectrometer software.^15,18^

#### 3.3.3 Spectrum correction and baseline adjustment

Fourier Transform converts the time signal into a spectrum, but this spectrum must be corrected for artifacts of the instrument or baseline correction. This step is especially needed when the sample or instrument causes the spectrum baseline (or starting line) to drift so that it hides and/or distorts salient peaks. Software usually has tools to correct these problems, with the aim of providing spectra that are a faithful representation of properties of the samples.^15^

#### 3.3.4 Spectral interpretation

Using the corrected spectrum, the molecular structure and composition of the material can be determined. Each peak in the spectrum correlates with a vibrational mode of the molecules in the sample, and by analyzing these, they can be identified to reveal what functional groups are present. Here, software tools are available to support this process (to match the observed spectrum with reference spectra present in databases) for identifying unknown compounds or confirming known substances.^21^

#### 3.3.5 Quantitative analysis

FTIR characteristics involve qualitative identification as well as quantitative analysis. If the concentration of one component in a mixture is known, then specific absorption bands are taken and their intensity marked. This needs meticulous calibration with the standards and consideration of parameters such as path length and sample thickness, which can influence absorbance.^22^

#### 3.3.6 Data storage and reporting

The processed data is stored digitally to enable retrieval, comparison, and sharing. Today, FTIR software development often integrates more expanded options in order to produce comprehensive reports with spectra alongside analysis results and method information that are critical for research as well as industrial applications.^23^

The successful application of FTIR in the analysis of inorganic materials is critically dependent on components of an FTIR spectrometer, sample preparation techniques, and data acquisition and processing methods. Every stage in the procedure, starting from sample preparation to the extraction of the spectrum at the end, requires special consideration for obtaining meaningful and accurate results.^24^

## 4 Applications of FTIR in inorganic material analysis

FTIR is an industrial technique that can be applied in many fields, including inorganic materials. It enables analysts to determine information related to the chemical makeup, chemical structure, and phase of the different active substances, among others. We will survey FTIR applications in chemical composition and structure identification, as well as crystalline and amorphous phases. Next, we focus on a few inorganic materials: oxides, silicates, and carbonates, to understand the FTIR setup we have described earlier here. When assessing molecular weight, ionization can take place before the identification of chemical composition and structure; however, this process can potentially destabilize certain bond types.^21^

### 4.1 FTIR application in inorganic materials based on their basic classification

Inorganic materials can be classified into several categories based on their chemical composition, bonding, and structure. In this paper, we described the FTIR application of some of these basic classifications.

#### 4.1.1 Oxides

Oxide compounds are made of oxygen and one or more other elements (usually metals). Both simple oxides (*e.g.*, SiO_2_, TiO_2_, and Al_2_O_3_) and complex mixed oxides (*e.g.*, spinels and perovskites) are being analyzed by FTIR spectroscopy. The main characteristics detected in the FTIR spectrum of oxides are metal–oxygen stretching and bending vibrations. For instance, in the FTIR spectrum of titanium dioxide (TiO_2_), there are strong absorption bands in the 400–800 cm^−1^ range due to Ti–O stretching vibrations. However, the disparities in these bands, which may be associated with crystallization, can differentiate between polymorphs of TiO_2_ like anatase and rutile.^25,26^ And metal–oxygen (M–O) stretching: 400–1000 cm^−1^. Si–O–Si asymmetric stretching (silica): 1050–1250 cm^−1^.^26,27^

#### 4.1.2 Carbonates and bicarbonates

**Fig. 3:**
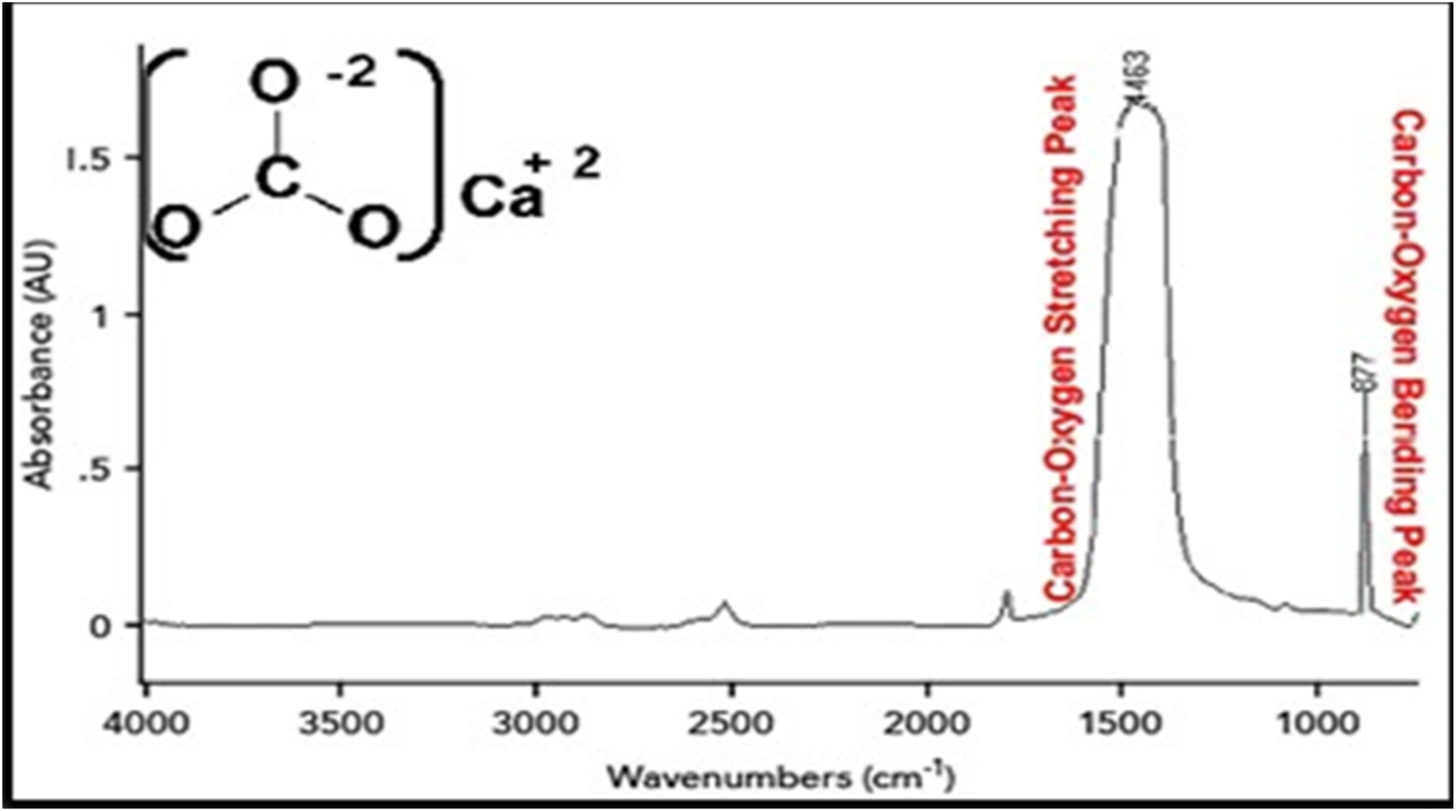
FTIR spectrum of CaCO_3_.^31^

The abundance of such carbonate minerals calcite (CaCO_3_), dolomite, CaMg(CO_3_)_2_ in sedimentary rocks affects carbon cycling and environmental processes. Their behaviour informs the prediction of geological history, carbon sequestration, and carbonate reservoir making. The different absorption bands of the carbonate ion (CO_3_^2−^) in FTIR are used to distinguish carbonate minerals. Calcite usually exhibits significant absorption bands around 1400 cm^−1^ (asymmetric stretching) and 870 cm ^−1^ (out-of-plane bending).^28^ Using FTIR to identify various carbonate minerals in rock samples could help to understand diagenetic processes that modify the fabric characteristics of a rock, including recrystallization or dolomitization (Fig. 3). Such information is critical for oil and gas exploration, environmental monitoring, and other geological inquiries.^29,30^

#### 4.1.3 Phosphates

**Fig. 4:**
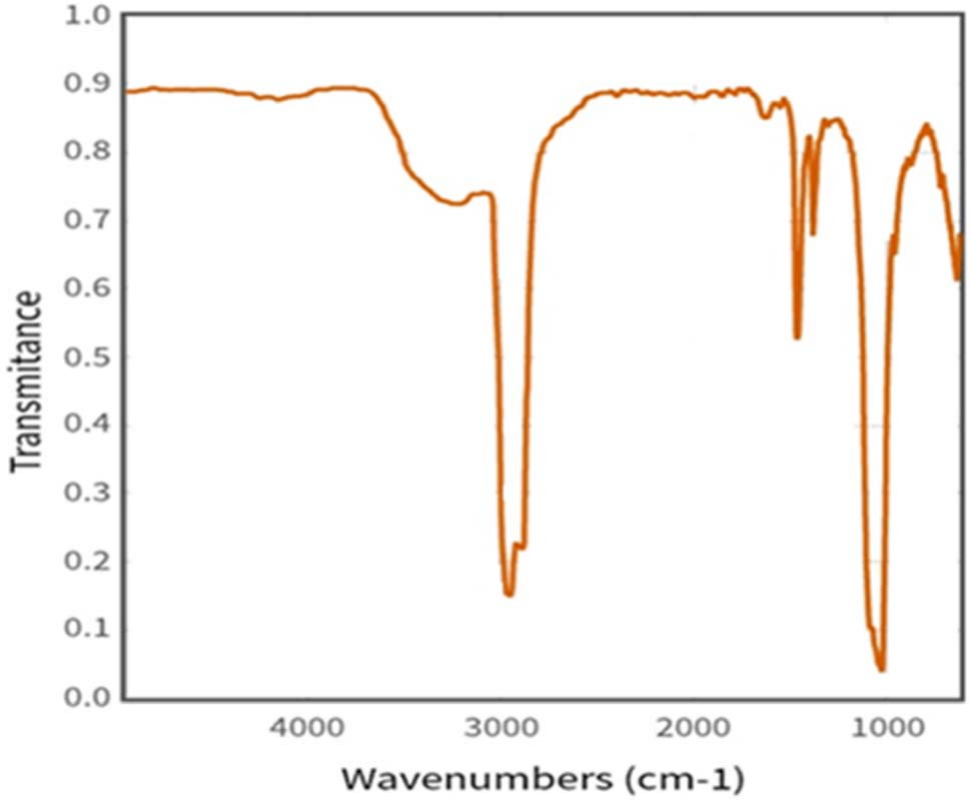
FTIR spectra of calcium phosphate, tribasic.^30,36,37^

Mainly, Phosphate materials contain PO_4_^3−^ groups and are found in minerals like apatite (Ca_5_(PO_4_)_3_(OH)). And their detection wavelength is P–O bending vibrations: 400–600 cm^−1^ (Fig. 4). P–O asymmetric stretching: 1000–1200 cm^−1^. P–O symmetric stretching: 900–950 cm^−1^.^32–35^

#### 4.1.4 Silicates

**Fig. 5:**
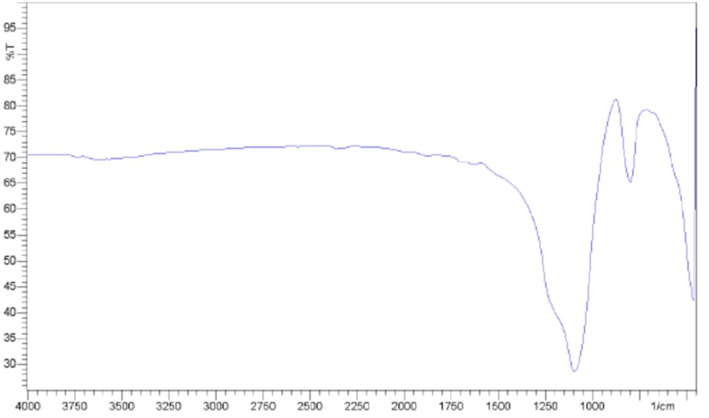
FTIR spectra of silica.^38,41,42^

Silicates are the most abundant group of minerals on Earth; they consist of silicon and oxygen, often combined with other elements such as aluminium, magnesium, and iron. The most characteristic features of the silicates in FTIR spectra are Si–O stretching and bending vibrations. Position and shape of the Si–O absorption bands inform on silicate structure, for example, the spectrum of a sheet silicate (*e.g.*, mica) will look very different from that of a chain silicate (*e.g.*, pyroxene) or even a framework silicate (*e.g.*, quartz) (Fig. 5). FTIR detection of various forms of Si–O bonding (bridging *vs.* non-bridging oxygens, *etc.*) is important in understanding some aspects of silicate structure and reactivity. Si–O–Si asymmetric stretching: 950–1250 cm^−1^. Si–O bending vibrations: 400–800 cm^−1^.^38–40^

#### 4.1.5 Sulphates and sulphides

**Fig. 6:**
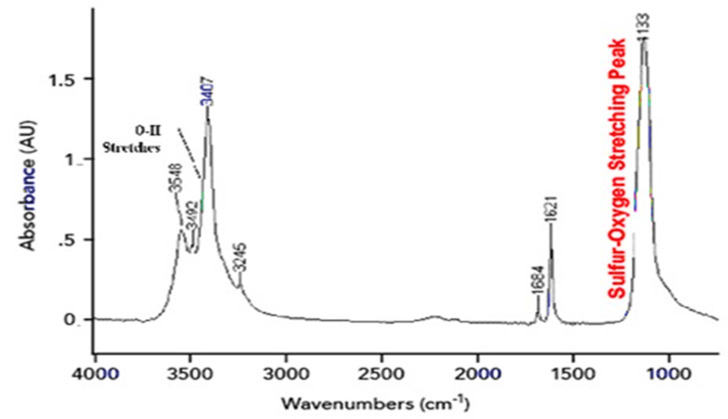
FTIR spectrum of calcium sulfate dihydrate (gypsum).^29,47,48^

Mainly sulphates (SO_4_^2−^) and sulphides (S^2−^) carrying materials like gypsum (CaSO_4_·2H_2_O) and pyrite (FeS_2_). S=O stretching in sulphate is 1000–1200 cm^−1^ (Fig. 6). And SO_4_^2−^ bending mode: 600–650 cm^−1^. Metal–sulfur (M–S) stretching: 250–450 cm^−1^.^31,43–46^

#### 4.1.6 Nitrates

In inorganic material, a large quantity of nitrates is present. Mainly, KNO_3_ and NaNO_3_ contain NO_3_^−^ groups. This group absorption band in FTIR is N–O stretching: 1300–1500 cm^−1^. Nitrate bending modes: 700–850 cm^−1^.^49^

#### 4.1.7 Halides

Halides contain mainly halide anions like Cl^−^, Br^−^, and F^−^, and these anions are found in salts like NaCl, KBr, and CaF_2_. This halide-metal absorption bend is (M–Cl, M–Br, M–F): 150–500 cm^−1^.^3,50^

#### 4.1.8 Borates

Mainly, we found Borates in BO_3_^3−^ or BO_4_^5−^ groups, as in borax (Na_2_B_4_O_7_·10H_2_O). And their absorption band is B–O stretching: 1200–1500 cm^−1^. B–O–B bending vibrations: 500–800 cm^−1^.^51–55^

#### 4.1.9 Hydrotalcite

Layered double hydroxides (LDH) or hydrotalcite-like compounds belong to the anionic clay family. One of the ways to identify the structures of LDH and intercalated anions between LDH layers is the FTIR technique. In all FTIR spectra of prepared LDH, a broad absorption band was observed around 3480 cm^−1^ with a shoulder band around 3000 cm^−1^, which was related to OH stretching vibration.^56,57^

#### 4.1.10 Peroxides

FTIR is widely used to identify peroxides through the O–O stretching vibration, typically observed near 877 cm^−1^. This band may shift depending on the sample environment, such as matrix interactions or hydrogen bonding.^58^

For different peroxide compounds, the O–O stretching range generally lies between 845–875 cm^−1^, with variations based on structural differences.^59^ Studies with isotopically labeled hydrogen peroxide (D_2_O_2_) show band shifts that help interpret bond dynamics.^60^

#### 4.1.11 Azides

Azides are known for their linear N_3_^−^ ion structure, and FTIR spectroscopy serves as a key method to detect this functional group by its strong asymmetric stretching vibration around 2000–2150 cm^−1^.^61^ In simple inorganic azides such as sodium azide, the N_3_^−^ stretch is typically seen near 2040 cm^−1^, serving as a reliable spectral marker for this functional group.^62^ The frequency of the N_3_^−^ vibration can shift slightly depending on the metal ion or the surrounding chemical environment. For instance, metal azides may exhibit variations in the N_3_^−^ band due to coordination effects or hydrogen bonding.^63^ Moreover, azide ions show sensitivity to solvent and matrix effects, as demonstrated in micellar systems where the stretching band position varied with different surfactants.^64^

#### 4.1.12 Perchlorates and chlorates

Perchlorate (ClO_4_^−^) and chlorate (ClO_3_^−^) ions show distinct Cl–O stretching bands in FTIR, making them easy to identify. Perchlorates typically exhibit strong bands around 1100–1000 cm^−1^, while chlorates appear near 950–1000 cm^−1^.^65^ The position of these bands can vary slightly depending on the associated cation and hydration level.^66^ FTIR is especially useful for distinguishing these species in complex mixtures such as propellants, water samples, or oxidizer blends.^31^

### 4.2 Identifying chemical composition and structure

FTIR spectroscopy provides suitable signals for characterizing the chemical composition and molecular structure of inorganic materials. It is sensitive to distinct vibrational modes of specific types of chemical bonds. Infrared light interacts with a sample to be analyzed, where it absorbs at frequencies matching the vibrational frequencies of the bonds in the molecules. The spectrum thus obtained represents a unique characteristic of the material, which can be used for identifying certain functional groups and therefore chemical constituents.^15^

### 4.3 Identification of the functional group

The characteristic absorption bands of different functional groups, such as hydroxyl (O–H), carbonate (CO_3_^2−^), silicate (Si–O), and phosphate (PO_4_^3−^) groups, were exhibited in FTIR spectra. Scientists can identify the functional groups for a material by studying the position, intensity, and shape of these bands. Bands observed at or near 1400 cm^−1^ may indicate the presence of carbonate ions, and bands between ∼1000–1200 cm^−1^ are common indicators of silicate structures.^5,87^

### 4.4 Bonding environment

FTIR can further give information related to the bonding environment of the elements within a material. This may be the splitting or shifting of absorption bands with different coordination states of the metal ion, *i.e.*, tetrahedral *vs.* octahedral coordination. This is especially true throughout the investigation of elaborate oxides and minerals in which the close-by environment surrounding metal ions is a determining factor within the properties of a fabric.^68^

### 4.5 Polymeric structures

FTIR can also provide useful information in complexes with polymeric structures (silicates and phosphates) by helping clarify the length of the chain and the connectivity of the units within the polymer. Position and intensity of particular absorption bands indicate the existence of bridging oxygen atoms (Si–O–Si linkages) or non-bridging oxygens (Si–O^−^).^38,69^ Polymers display unique infrared absorption spectra typically corresponding vibrantly to characteristic modes of constituent functional groups within their molecular structure. Analysis of solid-state polymers like films and fibers has become fairly routine with modern FTIR configurations, especially those with ATR accessories. Key spectral regions provide direct evidence of backbone structures and side chains and potential cross-linking through C–H stretching around (2850–2950) cm^−1^ and carbonyl stretching (C=O) stretching (1700–1750 cm^−1^), O–H and N–H stretching (3200–3600 cm^−1^), and C–O–C stretching (1000–1300 cm^−1^). FTIR is routinely employed to differentiate homopolymers from copolymers and evaluate the extent of some obscure functionalization pretty accurately. Analysis of band shape intensity and position enables a semi-quantitative assessment of crystallinity in semicrystalline polymers like polyethylene quite effectively. Advanced developments, including two-dimensional correlation FTIR and FTIR microspectroscopy, enabled spatially resolved chemical imaging of polymer blends and multilayer systems, facilitating myriad studies of phase separation behavior at interfaces of various polymer systems. FTIR is very useful for keeping track of oxidative and hydrolytic reactions because it can pick up signs of different functional groups, such as carbonyl and peroxide groups. When we can combine FTIR with chemometric methods like PCA (Principal Component Analysis) or PLS (Partial Least Squares), we can get better results at measuring things, especially when we look at additives, residual monomers, and polymer–filler interactions.^70,71^

### 4.6 Quantitative analysis

FTIR can also be used quantitatively in addition to qualitative identification. This allows for concentration determination of specific components in a mixture, based on known standards calibrated into the spectrometer, and the absorbance of characteristic bands. IR spectroscopy is essentially a quantitative analysis, built on the principle that absorption of any infrared light at a certain wavelength varies proportionally with the number of absorbing species in the material. This is especially helpful in the identification of material purity or the abundances of a specific phase in a composite. The intensity of specific absorption bands associated with certain bonds or functional groups, such as metal–oxygen bonds, is used in the analysis of mineral samples. Such functionality is particularly important in industries such as materials science and environmental monitoring, where it can be critical to know the exact content of a material. The multifaceted and critical approach of FTIR for both qualitative and quantitative determination provides significant information for the study of inorganic materials.^72^

### 4.7 Phase identification of inorganic materials by using FTIR

FTIR can be utilized with high efficacy in order to discriminate between the different phases of inorganic materials. In particular, it has been advantageous in characterizing and distinguishing polymorphs, different structural forms of a compound. Calcium carbonate, for instance, occurs as calcite, aragonite, and vaterite, but each has different structural features. Subtle differences can be observed in the vibrational modes of individual polymorphs measured with FTIR, permitting accurate phase identification.

That is important in mineralogy, materials science, and industrial processing since the physical properties of a material (*e.g.*, mineral) can be very different from one phase to another. The identification of such phases through FTIR allows scientists and engineers to gain a better understanding of the material behavior, optimize processing conditions, and ensure the required properties in final products. FTIR has thus remained an integral tool in the study and use of inorganic materials over a broad range of industries.^28,29^

#### 4.7.1 Crystalline phases

Atoms or ions in crystalline materials are arranged in a regular, repeating pattern. This results in sharp and well-defined peaks at specific wavenumbers corresponding to the vibrational modes of the crystal lattice in the FTIR spectrum. As a representative example, the FTIR spectrum of a silicate that exists in a crystalline structure will show sharp bands corresponding with the stretching and bending modes of the Si–O bonds contained within the silicate structure.

Based on the position and intensity of these bands, details regarding the crystal structure, bonding type, and symmetry can be obtained. As an example, subtle differences in FTIR spectra allow for the differentiation of various polymorphs of a mineral like quartz and cristobalite, both of which are forms of SiO_2_.^9^

#### 4.7.2 Amorphous phases

Amorphous materials do not have a long-range order, which means that their atoms or ions are arranged more randomly. As a result, FTIR spectra exhibit broader and less well-defined absorption bands due to this lack of order. It is referred to as broadening due to less well-defined vibrational modes in the disordered structure, which provides a range (not an exact value) of similar vibrational frequencies.^73^

FTIR is especially applicable for glasses and other amorphous materials. For instance, a silicate glass might show Si–O stretching vibrations as a broad band with a maximum near 1100 cm^−1^, as opposed to sharp peaks that would be observed in crystalline silicate [emphasis added]. The spectra can then be compared to that of the same material in a crystalline form, enabling researchers to calculate the extent of disorder and type of bonding within an amorphous material.^73,74^

#### 4.7.3 Phase transitions

FTIR can also be used to monitor transitions between the crystal and amorphous phase. For instance, the FTIR spectrum evolves as bands sharpen and new peaks emerge to highlight the crystallization of a glass. These changes can help us understand the structural modifications taking place in the material during the phase transition from an amorphous to a crystalline state.^75,76^

### 4.8 Surface analysis

FTIR is a powerful tool for the study of inorganic surfaces and coatings. It provides information on the composition of the surface in great depth, meaning that it can be used to study interactions with a molecular surface and how surfaces change during chemical reactions, corrosion, or exposure to other environmental factors.

For example, FTIR can identify the character of functional groups or chemical bonds adsorbed on surfaces of metal oxides or other coated materials, indicating how such characteristics specify their reactivity and stability as well as performance. Such surface analysis is crucial in catalysis, corrosion science, and materials engineering since this information enables a better tuning of the surface properties, thus leading to improved material performance and lifetime. FTIR serves as an important tool for the investigation and design of next-generation inorganic materials by allowing non-destructive probing of their surface properties.^47,49,77,78^

### 4.9 FTIR analysis of ceramics and glasses

Ceramics and glasses are important constituents in many applications, ranging from construction to electronics. FTIR is widely used for the study of their composition and structure, which are important factors for their property and performance optimization.

#### 4.9.1 FTIR analysis of alumina ceramics

Alumina (Al_2_O_3_): a common ceramic material used for its hardness, thermal stability, and electrical insulating properties. The phase composition and purity of alumina ceramics play an essential role in their performance for many industrial applications. FTIR is applied to determine the phase composition of alumina ceramics, and it can be conducted through the detection of some characteristic absorption bands related to the specified crystal form (morphology) of alumina. In fact, the spectrum of α-alumina exhibits sharp bands in the region of 600–800 cm^−1^ associated with Al–O stretching vibrations in the crystal lattice. FTIR spectra of the ceramic sample can be compared with published (standard) ones from the literature, enabling one to examine how pure an α-alumina phase is and whether or not any other phases, such as γ-alumina or unreacted (non-flocculated) alumina, are still present in this form. Such information may play an essential role in quality control and property definition of the ceramic for specific applications.^79–81^

#### 4.9.2 FTIR analysis of silicate glasses

Silicate glasses, like those found in window panes, optical fibers, or glass containers, are oxide-based amorphous materials with a structure primarily made of silicon dioxide (SiO_2_) and different modifiers, including sodium oxide (Na_2_O) or calcium oxide (CaO). The composition and properties of these glasses vary according to the species and concentration of modifiers. An evaluation of the silicate network is achieved by FTIR spectroscopy, focusing on Si–O stretching and bending vibrations. For a silicate glass, the spectrum usually contains wide bands in the 1000–1200 cm^−1^ region assigned to Si–O–Si stretching and into 400–500 cm^−1^ for Si–O–Si bending after.^38^ These bands can be examined to determine the polymerization level of the silicate network and how different modifiers affect the glass structure. Understanding the physical nature of these properties, which correlates with their resistance to breaking, chemicals, and optics, guides the development of glasses in specific tailored property ranges for particular applications.^41,78,82^

### 4.10 Characterization of minerals using FTIR

Minerals are naturally occurring inorganic substances that make up the rocks and soils. FTIR is widely used for mineralogical characterization and provides fundamental insight into material composition and structure in geological and materials science studies.^69^

#### 4.10.1 Characterization of clay minerals by FTIR

Clay minerals, including kaolinite, montmorillonite, and illite, are essential in soils and sediments. It is their characteristics that affect the behaviour of soil, including water retention, cation exchange capacity, and reactivity. Identification of these minerals is important to soil mechanics, fertility, and interaction with environmental aspects. Identification of clay minerals by FTIR: overview based on the assignment of characteristic OH stretching vibrations and Si–O bending modes. The example includes kaolinite strong absorption bands at 3700–3620 cm^−1^ (OH stretching) and 1030 cm^−1^ of Si–O stretching.^83^ Fourier Transform Infrared spectroscopy (FTIR) allows geologists to accurately determine which clay minerals are present in a soil or sediment sample—key components for uncovering the origins, weathering history, and agricultural suitability of soils and sediments. It helps environmental assessment purposes as well because it tells how the soil reacts to a particular pollutant.^83,84^

### 4.11 The FTIR in the analysis of corrosion products in inorganic materials

In industries that use metals and alloys for construction exposed to any corrosion environment, treatment development holds paramount importance. Knowledge of the nature and morphology of corrosion products is critical in controlling or suppressing corrosion. FTIR analysis can be performed non-destructively on these products, making it useful for providing insights into corrosion.^85^

#### 4.11.1 FTIR analysis of iron corrosion products

Corrosion of iron and steel structures in humid atmospheres, *e.g.*, bridges, pipelines, and industrial objects, results in rust (iron oxides and hydroxides). Detecting the exact types of corrosion products is crucial for measuring the severity of corrosion and choosing the most suitable protection and repair techniques. An abstract overview of the transmission Fourier transformed infrared spectroscopy (FTIR) technique is presented, together with examples of its use for analysing corrosion products formed on iron surfaces through identifying absorption bands characteristic of various iron oxides and hydroxides. Absorption bands near 800 cm-owing to the presence of goethite (α-FeOOH), can be found in the spectra of in order and around 570 cm-can be associated with magnetite (Fe_3_O_4_). Analysis of FTIR aids in knowing the phases of iron oxides available on the corroded surface and assists in providing information on different environmental conditions, which lead to corrosion. Understanding these insights can help develop efficient corrosion inhibitors, coatings, and maintenance guidance for the preservation of iron and steel structures.^86–89^

#### 4.11.2 FTIR analysis of copper corrosion products

Copper and its alloys are used in the manufacture of electrical wiring, plumbing, and architectural features because of their corrosion resistance. Copper, however, is not stable in all environments and will corrode to produce products such as patina (basic copper carbonate) or cupric oxide. Knowing about these products is one of the key aspects to maintaining the visual and functional properties of the copper surface. FTIR is used to detect the characteristic absorption bands of different corrosion products deposited on the surface of copper, such as cupric oxide (CuO) and copper carbonate (Cu_2_CO_3_(OH)_2_). For instance, the bands of cupric oxide appear around 530 cm^−1^. While FTIR analysis allows for identifying in detail the different corrosion products appearing on copper surfaces, these functional groups can then be used to show the effectiveness of various protective coatings and also of environmental factors such as humidity, pollution, and acid rain. These insights are important for the conservation of copper heritage sites, increasing the service life of Cu alloys, and designing new corrosion-resistant alloys.^85,87,90^

### 4.12 FTIR application for the detection of impurities, contaminants, and verification of purity in inorganic materials

Fourier Transform Infrared spectroscopy (FTIR) is a powerful and versatile analytical technique extensively used for detecting impurities and contaminants and for verifying the purity of inorganic materials. This method is based on the principle that distinct chemical compounds absorb infrared radiation at characteristic wavelengths, producing unique spectral fingerprints that can be used for qualitative and quantitative analysis. FTIR is capable of identifying both organic and inorganic impurities at trace levels, making it essential in applications where material quality, safety, and performance are critical—such as in the pharmaceutical, construction, electronics, and manufacturing industries.^1,2,67^ Even minor deviations from reference spectra can indicate the presence of unwanted substances that may compromise the structural integrity or functional behavior of materials like metals, ceramics, or minerals. For example, trace water or carbon-based contaminants in metal alloys can accelerate corrosion and reduce mechanical strength over time. FTIR's wide detection range allows for the identification of organic impurities (*e.g.*, oils, resins) introduced during manufacturing or handling, as well as inorganic contaminants such as heavy metals, salts, or process residues. Furthermore, FTIR plays a crucial role in environmental monitoring by detecting pollutants in soil, water, and air—enabling early intervention before these contaminants adversely affect human health or ecosystems. Its non-destructive nature, high sensitivity, and rapid analysis make FTIR a critical tool for quality control and environmental safety. By ensuring the detection and characterization of impurities, FTIR contributes significantly to maintaining the purity, consistency, and performance of inorganic materials across a wide range of applications.^91^

### 4.13 Study of hydration and dehydration processes in inorganic materials using FTIR

Fourier Transform Infrared spectroscopy (FTIR) which is used widely to study the hydration and dehydration mechanisms of many other materials such as cement, gypsum, and hydrous minerals. The processes of addition and removal of water molecules are particularly important for many materials, such as those frequently used in construction and industrial applications, because they significantly influence their physical–chemical properties. Through monitoring changes in the vibrational frequencies of chemical bonds – specifically for O–H (oxygen–hydrogen) stretching vibrations associated with water—FTIR sheds light on these transformations.^92^

Hydration is a chemical reaction between water and the material, generally under the condition of deep binding and absorption, to cause a great change in performance. They are usually observed in substances; on the other hand, during the hydration process, cement reacts with water. Cement hydration is an important process in construction, which produces a hardened structure. FTIR enables researchers and engineers to monitor this process in real time by observing discrete IR absorption bands characteristic of O–H stretching. The composition of the spectrum changes as the cement hydrates, which contain critical information for stages in hydration, reaction kinetics, and stability of the hydrates.^92,93^

Gypsum, a major construction material, also goes through a similar hydration–dehydration process. It is a hydrous mineral, which means that its crystals contain water. Gypsum loses its water molecules when heated, in a process called dehydration, and think of it as changing into another mineral, anhydrite. When anhydrite takes up water, it reverts to gypsum. FTIR analysis allows transitioning from a dry to an oriented structure, which is detected by shifts in the O–H stretching vibrations associated with water that is either bound or absent. Such data improves the understanding of gypsum behaviour during different processing routes, as well as requiring different environmental conditions that subsequently enhance the performance of various end-products such as plaster and drywall.^93^

Aside from construction materials, FTIR has also been employed to investigate the dehydration and hydration behaviour of other hydrous minerals. These minerals that are found in geological settings readily adsorb water and can lose or gain water with changes in environmental conditions, such as temperature and pressure. This increase in halite and other minerals, where the shift is instrumentally determined through FTIR (Fourier Transform Infrared spectroscopy) spectra analysis, may help scientists better understand how these minerals behave under those circumstances. As an example, clay minerals are a focus for studies related to their ability to hold or release water, and have applications in fields such as soil science and environmental monitoring.^93^

The capability of FTIR to monitor the hydration and dehydration processes also gives significant insights into this permanent behavior, as water diffusion is a fundamental driver of long-term durability for all materials. This is useful because the technique detects local changes in materials at the molecular level, providing insight into what is happening topically. In addition, FTIR is an inherently non-destructive technique that can be utilized to analyze samples without significantly changing the physical and/or chemical properties of a sample; therefore, it is particularly suitable for studying ongoing processes such as hydration/dehydration. FTIR helps in increasing our horizons on the hydration and dehydration process of materials. FTIR assists in enhancing the properties of construction materials, is used in environmental studies, and offers information on natural geological processes by tracking variations in moisture content and linked chemical bonds. Because of its precision and versatility, it becomes an important instrument when both hydration and dehydration processes are crucial, either in research or industrial applications.^92,94,95^

### 4.14 Catalysis research in inorganic materials using FTIR

The vital role of Fourier Transform Infrared spectroscopy (FTIR) in catalysis, revealing the surface characteristics of catalysts and the reactions occurring on their surfaces. Catalysts (metal oxide, zeolite, *etc.*) are extensively used in industries to accelerate chemical reactions without being consumed during the reaction cycle. The novel catalyst is likely to be a function of its surface properties so as to interact efficiently with the reactants and assist in generating the end product(s). FTIR is a key technique in this project as it enables direct examination of adsorbed molecules and reaction intermediates on the catalyst surface, providing vital information regarding both the reaction mechanisms and catalyst work. FTIR serves many purposes in the research on catalysis, but one of the main ones is studying how molecules interact with the catalyst surface. During a chemical reaction that occurs with the presence of a catalyst, reactant molecules will usually adsorb onto the surface of that catalyst. FTIR permits detection of these adsorbed species through their characteristic absorption bands due to the associated vibrational modes. Based on the resulting spectrum, they are able to identify which molecules are present, their orientation upon the surface, and how tightly each molecule is bound. It helps in studying the initial phase of a reaction that is important for enhancing catalyst productivity.^96,97^ FTIR also affords useful information on reaction intermediates, which are ephemeral species that exist during a chemical reaction but do not appear in the final products. Such intermediates can provide important clues as to the manner in which a catalyst works—that is, the stepwise pathway it takes to stimulate a reaction. By monitoring the time evolution of these intermediates, FTIR allows researchers to trace the reaction pathway and glean key insights into the details of the catalytic mechanism. This mechanism can be translated directly into a catalyst design (and optimization), as it informs one which surface features or conditions would best favor the desired reaction.^98^

FTIR is especially helpful in exploring metal oxide or zeolite-based catalysts. Metal oxides are widely used as catalysts in industrial applications such as chemical and fuel production, as well as environmental remediation.^99^ Metal oxides are generally layered materials that offer active sites to chemical reactions on their surfaces, and FTIR can characterize the adsorption of reactants and the formation of intermediates on them.^100^ This data is necessary to further tailor the composition and structure of the material to enhance its activity.^101^ Another type of catalyst that is often studied by FTIR is zeolites, which are crystalline porous materials. They have an unusual property to hold molecules in their pores like a needle, and fine-tune the conditions where catalytic reactions can happen.^102^ Therefore, FTIR also enables researchers to gain insight into the mechanisms by which reactants penetrate into the pores, interact with active sites in their interior, and products are formed and desorbed.^103^ Knowing how to use hotspots will help engineers design better zeolite catalysts for petrochemical refining and environmental protection.^104^ FTIR advances the science of catalysis in multiple dimensions—detailed information on surface interactions and reaction mechanisms.^105^ It enables scientists to determine the different functions of surface species in catalysis, facilitating optimal catalyst design for industrial processes where improved acceleration of reactions with reduced energy expense and cost are often desirable. FTIR can also be used *in situ*, where you can monitor surface changes during reaction time.^106^ FTIR is an important technique when it comes to studying heterogeneous catalysis and determining the surface properties of catalysts to unlock the mystery of how these catalysts operate. Such information is paramount to catalyst model design—allowing for more efficient chemical processes and industrial systems that make the most out of their respective catalysts.^100^ FTIR is one of the few techniques capable of studying adsorbed species and reaction intermediates, making it an ideal tool for understanding catalytic mechanisms.^106^

### 4.15 FTIR for inorganic material quality assurance

Verification of materials is one of the most basic requirements for the quality of products in many sectors, and it is essential for high-performance and trustworthy products. The Fourier Transform Infrared spectroscopy (FTIR) serves as an efficient analytical tool for inspecting their composition and purity in inorganic form. FTIR is an important element in ensuring quality control by offering specific details about the molecular structure and chemical bonds of a material. This is particularly critical in industries where damage to the material can lead to some serious problems, like electronics, construction, and pharmaceuticals.^15^

In the manufacturing world, uniformity of material quality is crucial as any defects can lead to product failure. Far-Infrared Transmission spectroscopy allows manufacturers to identify raw material compositions, test for contaminants, and verify that they pass minimum specifications. This works by creating an infrared absorption spectrum analysis of the material. All chemical compounds have a distinct spectral fingerprint with absorbance maxima, which absorb infrared light at specific wavelengths. FTIR is based on accurately comparing the spectrum of a sample against known reference spectra, establishing chemical identification, and catching traces of impurities.^107,108^ FTIR is particularly useful when trying to identify impurities in inorganic materials. Impurities will reduce the performance and lifespan of materials, especially in sensitive applications. In addition, some industries like electronics are negatively affected by the presence of impurities in semiconductors, metals, or ceramics that can hinder disruption of electrical properties within components, resulting in device malfunctions/failures. FTIR is used for the analysis of such materials and also identifies residuals by detecting minute quantities of contamination, resulting in high-quality materials used in electronic devices. This is beneficial to manufacturers as it produces consistent products, eliminating the risk of faulty products and legacy devices.^109^ Besides impurity detection, FTIR is also useful for checking the uniformity and reproducibility of materials. One prominent source of variability is during manufacturing, where raw materials can differ in composition or processing conditions, such that they cause different properties or characteristics to evolve through the life cycle. These variations can lead to products that do not function or work at expected levels and fall short of industry standards. Continuous monitoring of the material composition and adjustment, if necessary, is always a plus in FTIR so that manufacturing can maintain consistency. This information is used in real-time to prevent potential costly mistakes, while making sure that a product meets specifications.^110^ Likewise, FTIR aids in material quality assurance for the construction industry too. Construction depends on the strength of inorganic materials such as cement, concrete and glass that ensure the safety and durability of structures. These materials are also FTIR analyzed for their purity and composition, determining any presence of undesirable compounds that would affect the performance of these materials. FTIR helps create safer and more stable buildings by making sure that contaminants and impurities are not present in construction materials.^111^ FTIR is also used in the pharmaceutical and manufacturing industries for quality control, where high precision/accuracy of the results is necessary. Molecular impurities are commonplace in the pharmaceutical industry, and inorganic materials such as excipients and catalysts may be involved in drug manufacturing. According to FTIR, the materials to be incorporated into the formulation need to be tested for purity and overall composition to ensure that they meet the standards of stringent regulatory requirements without having an impact on the terminal product. These actions help to protect consumers and maintain the integrity of pharmaceutical manufacturers in upholding the safety and efficacy of medications.^111,112^ FTIR is an invaluable tool for quality assurance in various industries. It is an essential equipment for ensuring adherence to high production standards due to its capability of identifying contaminants, ensuring composition, and checking consistency. FTIR offers manufacturers insight into the materials being used, allowing businesses to reduce defects, enhance reliability, and aid in overall quality control. Within industries where the repercussions from even minute material imperfections can be catastrophic, FTIR assists in mitigating failures and also confirms that products possess optimal performance.^15^

### 4.16 FTIR analysis of metal–organic frameworks (MOFs)

**Fig. 7:**
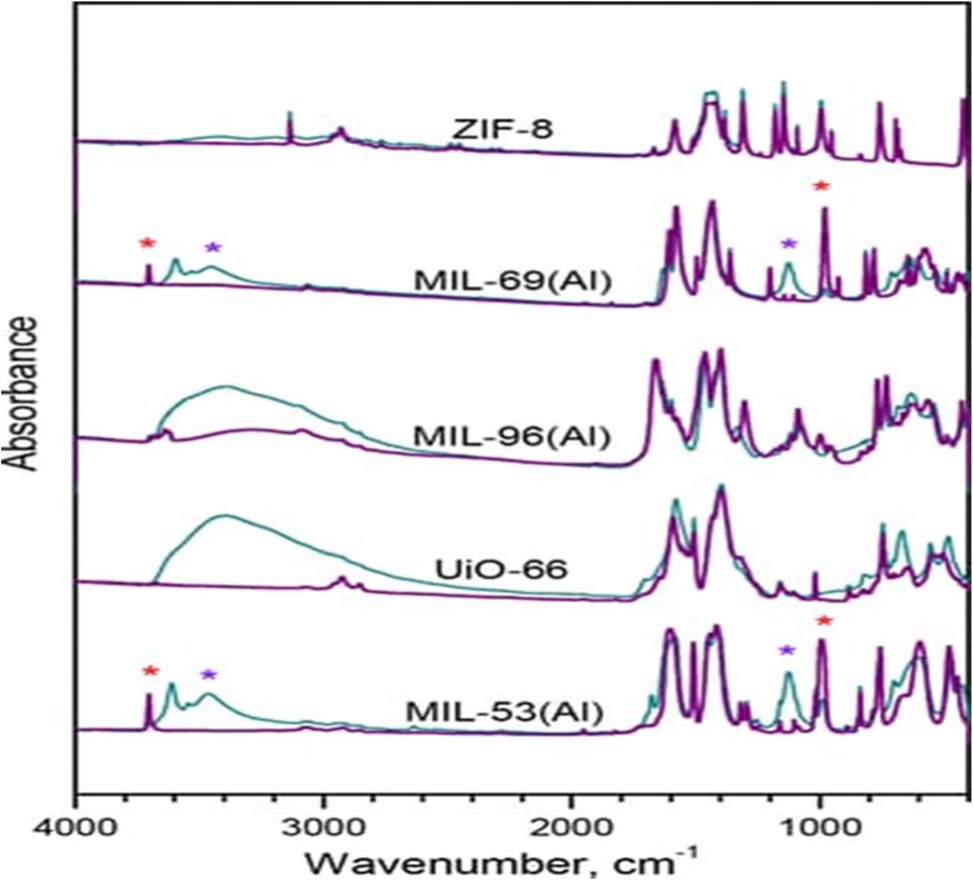
FTIR spectra of various MOF samples.^115,118,119^

FTIR spectroscopy plays an important role in the characterization of MOFs by identifying the functional groups involved in coordination and detecting changes during synthesis or post-synthetic modification. Specific vibrational bands corresponding to metal–oxygen, metal–nitrogen, and carboxylate groups are monitored to confirm successful MOF formation and to study their stability under different conditions. For example, in MOF-5 (Zn_4_O(BDC)_3_), the carboxylate group from terephthalic acid (BDC) is found to be coordinating to Zn^2+^ centers as seen in the appearance of asymmetrical –COO^−^ stretch at 1570–1600 cm^−1^ and a symmetrical stretching mode at 1360–1400 cm^−1^ replacing the free acid C=O stretch around 1700 cm^−1^. Analogously, in HKUST-1 (Cu_3_(BTC)_2_), the featured *p* carboxylate peaks are located at 1640 cm^−1^ (asymmetric) and 1370 cm^−1^ (symmetric) (accounting for framework fulfillment). The broad O–H stretch at 3400 cm^−1^ in MIL-101(Cr) is a signature that arises due to the uncoordinated water or –OH groups, and bound carboxylate stretches are seen at 1585 cm^−1^ and 1390 cm^−1^. Whereas the imidazolate linker C=N and C–N stretching vibrations are located around 1580 cm^−1^ and 1145 cm^−1^ for ZIF-8 Zn(2-methylimidazolate)_2_, respectively, while the Zn–N stretching mode appears at ∼420–440 cm^−1^.^113–116^ FTIR can also reveal defect structures within MOFs by detecting deviations in bonding environments, making it a valuable tool for materials engineering and catalysis research. For example, in defective UiO-66(Zr), when linkers are absent, Zr-nodes are bound to terminal hydroxyl or water ligands. These defects are usually characterized by a wide O–H stretching band in the ∼3670–3500 cm^−1^ region and additional Zr–OH bending vibrations in the 800–700 cm^−1^ range that are absent or greatly weakened in defect-free UiO-66. In the case of HKUST-1, the subtle collapse or defect of the framework results in a decrease in intensity of the symmetric and asymmetric carboxylate stretches (1370 cm^−1^ and 1640 cm^−1^*ca.*) and a reappearance of a well-defined peak ∼1700 cm^−1^, corresponding to the presence of free carboxylic acid moieties. Furthermore, ZIF-8 upon thermally or chemically decoupling exhibits a shifting or splitting of the C=N (1580 cm^−1^) and Zn–N (420–440 cm^−1^) stretching bands, indicating breaking of Zn–N bonds or linker substitution defects.^115,116^ Additionally, FTIR analysis allows the monitoring of guest molecule adsorption and desorption processes within MOF pores, providing insights into their potential as gas storage, separation, or sensing materials. For example, the adsorption of CO_2_ in MOF-74(Mg) is followed through its asymmetric stretching mode at ∼2343 cm^−1^, which increases in intensity when the CO_2_ is adsorbed, and decreases when desorbed under vacuum or mild heating. The shift and broadening of this band can also show the strength of interaction with open metal sites (Fig. 7). For H_2_O adsorption on UiO-66, we observe broad O–H stretching bands between 3200 and 3600 cm^−1^ and bending modes near ∼1640 cm^−1^, which are indicative of hydrogen bonding in the pores. When heated, the water is desorbed, and the bands in and reflect a considerable attenuation.^117–120^

### 4.17 FTIR characterization of cements and mortars

FTIR spectroscopy is a valuable tool for monitoring the chemical evolution of cement-based materials during hydration and aging. It enables the identification of key phases such as calcium silicate hydrate (C–S–H), calcium hydroxide (portlandite), ettringite, and carbonates formed during carbonation. The Si–O stretching band around 970–990 cm^−1^ is typically associated with C–S–H formation, while a sharp O–H stretching band near 3640 cm^−1^ is linked to portlandite.^121,122^ FTIR is also useful in tracking carbonation processes by observing the emergence of strong carbonate bands near 1420–1480 cm^−1^, indicative of calcite (CaCO_3_) formation in concrete exposed to CO_2_-rich environments.^123^ By studying these characteristic vibrational features, researchers can assess hydration degree, diagnose deterioration, and evaluate the effectiveness of additives and supplementary cementitious materials.^124^

### 4.18 FTIR use in nanoparticles and nanostructured inorganic materials

FTIR spectroscopy offers unique advantages in the characterization of inorganic nanoparticles and nanostructures, where surface chemistry dominates due to the high surface-to-volume ratio.

Unlike in bulk materials, vibrational features in nanomaterials are influenced by size effects, surface confinement, and quantum boundary conditions, which can shift peak positions and alter band shapes in FTIR spectra.^125,126^ In metal oxide nanoparticles such as ZnO, TiO_2_, or Fe_3_O_4_, FTIR can resolve not only intrinsic lattice vibrations (typically <600 cm^−1^) but also detect adsorbed surface groups such as hydroxyls, carboxylates, or sulfates, which are critical for stability, dispersibility, and functionalization.^127–129^ For example, the –OH stretching region (∼3400–3700 cm^−1^) provides insights into the degree of surface hydroxylation, which directly affects photocatalytic efficiency and reactivity.^130^ Furthermore, FTIR is instrumental in monitoring surface modification processes—such as ligand exchange, doping, or coating—through the emergence or disappearance of characteristic bands. For instance, FTIR tracking of citrate, PEG, or thiol binding groups on metal nanoparticle surfaces has been widely reported for biomedical applications.^131,132^ A critical strength of FTIR in nanoscale systems is its compatibility with *in situ* and *operando* configurations. This enables real-time observation of adsorption, desorption, or transformation reactions on nanoparticle surfaces, useful in catalysis, gas sensing, and energy storage research.^133–136^

In metal oxide nanoparticles such as ZnO, TiO_2_, and Fe_3_O_4_, FTIR can distinguish between lattice vibrations (typically below 600 cm^−1^) and surface-modified groups such as carboxylates, hydroxyls, and sulfates.^137,138^ A critical strength of FTIR in nanoscale systems is its compatibility with *in situ* and *operando* configurations. This enables real-time observation of adsorption, desorption, or transformation reactions on nanoparticle surfaces, useful in catalysis, gas sensing, and energy storage research.^139^

### 4.19 Monitoring thermal decomposition processes with FTIR

FTIR spectroscopy plays a significant role in studying the thermal decomposition behavior of inorganic materials. As materials are heated, chemical bonds break, gases are evolved, and phase transformations occur, which can be monitored in real-time using FTIR spectroscopy. Particularly when coupled with thermogravimetric analysis (TGA-FTIR), FTIR allows identification of gaseous decomposition products such as CO_2_, H_2_O, and SO_2_ released from solids.^140^ For example, the decomposition of calcium carbonate (CaCO_3_) into calcium oxide (CaO) and CO_2_ can be followed by the disappearance of carbonate vibrational bands (1400–1500 cm^−1^) and the appearance of gaseous CO_2_ signatures near 2350 cm^−1^.^141^ FTIR also allows the monitoring of thermal decomposition pathways in layered double hydroxides (LDHs), where distinct hydroxyl and carbonate bands diminish upon heating.^142^

### 4.20 FTIR in environmental monitoring of inorganic pollutants

FTIR spectroscopy has proven to be an effective technique for the identification and monitoring of inorganic pollutants in environmental samples such as soils, sediments, and waters. Many pollutants, including sulfates, nitrates, carbonates, and metal oxides, exhibit distinct vibrational fingerprints detectable by FTIR analysis. For example, sulfate ions display characteristic asymmetric stretching vibrations around 1100 cm^−1^, while nitrate ions show strong absorptions near 1384 cm^−1^.^91^ In addition, FTIR can detect changes in mineral surfaces caused by heavy metal adsorption, as observed through shifts in the vibrational bands of hydroxyl and carbonate groups.^143^ This technique has also been applied to monitor phosphate accumulation and metal–phosphate complex formation in contaminated soils and sediments.^144^

### 4.21 FTIR in the study of geopolymers and alkali-activated materials

Geopolymers and alkali-activated materials (AAMs) are inorganic binders formed by the reaction of aluminosilicate powders with alkaline solutions (Table 1). FTIR spectroscopy is a key technique for understanding the chemical evolution during geopolymerization. Characteristic Si–O–T (T = Si or Al) stretching bands, typically between 950–1100 cm^−1^, shift during polymerization, indicating the formation of a three-dimensional network.^115^ The decrease in intensity of hydroxyl bands (∼3400 cm^−1^) reflects the progress of dehydroxylation and condensation reactions.^145^ FTIR analysis enables the identification of reaction intermediates, gel phases (such as N–A–S–H or C–A–S–H), and the extent of alkali activation, providing valuable data for optimizing mechanical performance and durability.^146^

**Table 1:** FTIR spectroscopy absorption table

Wavenumber (cm^−1^)	Functional group/Compound	Possible bond type	Software name	Reference
1000–400 cm^−1^	M–O (metal–oxygen)	Stretching	OPUS, OMNIC	9, 26, 84 and 147
1000–850 cm^−1^	M=O (V=O, Mo=O)	Stretching	OPUS	9
700–500 cm^−1^	M–O–M (metal oxides, silicates)	Stretching	OPUS	9
700–400 cm^−1^	M–O (Fe–O, Co–O, Zn–O)	Stretching	OPUS	25
3700–3600 cm^−1^	Free O–H (adsorbed water, hydroxyls)	Stretching	OPUS, OMNIC	148–150
700–400 cm^−1^	Ti–O	Stretching	OPUS, OMNIC	26, 115, 133 and 134
1500–1400 cm^−1^	C–O (carbonates, CO_3_^2−^)	Asymmetric stretching	OPUS, OMNIC	29, 30, 36, 86, 154 and 155
900–880 cm^−1^	C–O (carbonates, CO_3_^2−^)	Symmetric stretching	OPUS, OMNIC	29, 30, 36, 86, 154 and 155
750–700 cm^−1^	CO_3_^2−^(Carbonates, CO_3_^2−^)	Bending vibration	OPUS, OMNIC	29, 30, 36, 86, 154 and 155
1200–1000 cm^−1^	S=O sulfate ion (SO_4_^2−^)	Stretching	OPUS, OMNIC	47, 48 and 156–158
650–600 cm^−1^	SO_4_^2−^Sulfate ion (SO_4_^2−^)	Bending	OPUS, OMNIC	47, 48 and 156–158
450–250 cm^−1^	M–S (metal–sulphur)	Stretching	OPUS, OMNIC	47, 48 and 156–158
1200–1000 cm^−1^	P–O phosphate PO_4_^3−^	Asymmetric stretching	OPUS, OMNIC	37, 159 and 160
950–900 cm^−1^	P–O phosphate PO_4_^3−^	Symmetric stretching	OPUS, OMNIC	37, 159 and 160
600–400 cm^−1^	P–O phosphate PO_4_^3−^	Bending	OPUS, OMNIC	37, 159 and 160
1250–950 cm^−1^	Si–O–Si (silica frameworks)	Asymmetric stretching	OPUS, OMNIC	38, 39, 41, 42 and 152
800–400 cm^−1^	Si–O (silicates, quartz, aluminosilicates)	Bending	OPUS, OMNIC	38, 39, 41, 42 and 152
500–150 cm^−1^	M–Cl, M–Br, M–F (metal–halide)	Stretching	OPUS	50
1500–1300 cm^−1^	N–O NO_3_^−^ (nitrates)	Stretching	OPUS, OMNIC	151
850–800 cm^−1^	N–O NO_3_^−^ (nitrates)	Bending	OPUS, OMNIC	151
1600–1200 cm^−1^	B–O borates BO_3_^3−^ or BO_4_^5−^	Stretching	OPUS, OMNIC	52–55
800–600 cm^−1^	B–O–B borates BO_3_^3−^ or BO_4_^5−^	Bending	OPUS, OMNIC	52–55
3500–3400 cm^−1^	N–H (primary amine)	Stretching	OPUS, OMNIC	161
2600–2550 cm^−1^	S–H (thiol)	Stretching	OPUS	162
600–400 cm^−1^	M–N (metal–nitrogen)	Stretching	OPUS, OMNIC	163 and 164
1100–1000 cm^−1^	M≡N (metal–nitride)	Stretching	OPUS, OMNIC	9 and 164
1100–1000 cm^−1^	M–N=N metal–azo ligand	Stretching	OPUS, OMNIC	9 and 164

## 5 Advantages and limitations of FTIR for inorganic materials

FTIR is a versatile analytical technique that can be used; however, the culture of FTIR has been dominantly focused on spectroscopy studies for inorganic materials.^153^ While it has multiple pros that make it one of the most essential techniques in material science, there are also cons that should be kept in mind while interpreting results. The following section will summarize the pros and cons of FTIR with respect to analyses of inorganic materials.^165^

### 5.1 Advantages of FTIR for the analysis of inorganic materials

Material science community prefers FTIR spectroscopy due to few reasons such as non-destructive, sensitive, and versatile nature of FTIR spectroscopic measurement. Some of the major benefits include.

#### 5.1.1 Non-destructive analysis

FTIR is non-destructive, and this is one of its greatest benefits. FTIR is non-destructive—unlike some analytical methods, no sample preparation, changing, or destroying the samples occurs. In fields like art conservation, archaeology, and heritage science, this is particularly vital since the integrity of the sample must be preserved.^166^

#### 5.1.2 Sensitiveness to molecular structure

FTIR sensitivity to the molecular structure of materials permits the detection of specific functional groups and bond types. The high sensitivity of this method makes it possible to achieve a detailed characterization of the chemical composition and molecular architecture of inorganic materials. FTIR, for example, can distinguish between different forms of the same mineral (*e.g.*, variations in polymorphs of (SiO_2_) based on slight variation in their spectra).^167^

#### 5.1.3 Broad applicability

FTIR is an inexpensive method that has been widely applied to many ranges of inorganic materials such as ceramics, glasses, minerals, oxides, and corrosion products. And the fact that it can be used to study both crystalline and amorphous phases makes it a powerful technique in material science. Additionally, FTIR is applicable for solids, liquids, and even gases.^168^

#### 5.1.4 Rapid and efficient data acquisition

One of the best applications for FTIR is in high-throughput situations due to its rapid data-acquisition capabilities. Some are based on Fourier transform algorithms, which permit the simultaneous measurement of all wavelengths in the infrared at once, and their signal processing relays rapid collection of data. This is rare in the industrial field, where many samples are required to be analyzed over a limited time.^169,170^

#### 5.1.5 Minimal sample preparation

FTIR usually requires little sample preparation compared to other analytical methods. In the case of many inorganic materials, the samples can be analyzed without any prior preparation (*e.g.*, in solid state: powders, thin films, or even bulk materials). This ease of use shortens the overall sample preparation time, reduces costs, and decreases the chance for contamination or other changes to the sample.^168,171^

#### 5.1.6 Quantitative and qualitative analysis

Methods of FTIR qualitative and quantitative analysis. FTIR is widely used for the identification of certain functional groups or compounds; however, the amount and concentration of these components in mixtures can also be quantified using FTIR. The amount of certain stuff inside the sample could be quantified with high accuracy using calibration curves and known standards.^172,173^

#### 5.1.7 Environmental and *in situ* studies

A FTIR is an *in situ* technique that can be accomplished under many environmental conditions, including high temperature and pressure. Thus, it makes it very versatile for investigating materials in bulk or under conditions that represent their actual applications. FTIR can be employed to investigate the growth of corrosion products on metallic surfaces *in situ*, which may yield information on corrosion mechanisms and approaches for protection.^26,91^

### 5.2 Disadvantages and limitations of FTIR spectroscopy

Advantages and disadvantages of FTIR in the analysis of inorganic materials. While not without proven positive attributes, FTIR has certain limitations that pose challenges for its application towards certain types of substances or specimen types. Being aware of these limitations is key to interpreting the data correctly, as well as to knowing when FTIR will or will not give you useful information in an analysis.

#### 5.2.1 Spectral interpretation difficulties

FTIR spectroscopy has a few challenges, like complex spectra interpretation. The absorption bands pertaining to different functional groups often overlap in many situations, and as a result, one cannot assign particular bands to distinct chemical species. Such artifacts can complicate the determination of a sample composition, most particularly when the mixtures are complex or where the target compound is present at low concentrations within the material.^97,174,175^

#### 5.2.2 Limited sensitivity to some inorganic materials

FTIR is sensitive to many inorganic materials but is insensitive in some cases, particularly for ionic and covalent bonds that do not change dipole moment during vibration. As an example, which will be relevant to many inorganic systems, homonuclear diatomic molecules (*e.g.*, O_2_ or N_2_) do not yield an FTIR signal because they do not possess a permanent dipole moment. In the same way, a few other crystalline materials show weak or poor spectra, making it difficult to study.^13,176^

#### 5.2.3 Challenges with sample preparation

FTIR is a technique that does not usually require excessive sample preparation, but there are some inorganic materials that could make the process more difficult or lead to results in terms of spectra quality. As an example, in the case of strongly reflecting or difficult-to-penetrate materials (*e.g.*, metals, some ceramics), owing to the scattering effect, the signal intensity is low or the spectra are distorted. In such instances, specific sample prep methods (such as thin films or ATR accessories) may be required to generate usable data.^168,171^

#### 5.2.4 Interference from water and atmospheric gases

FTIR spectra are influenced by the presence of water vapor and carbon dioxide in the atmosphere, which are strongly absorbed in the infrared region. Such interferences may hide important spectral information or result in baseline distortions, which make the analysis difficult. Even in modern FTIR instruments that come fully equipped with purging systems to get rid of atmospheric gases, these interferences could still be an issue in some cases.^98,177^

#### 5.2.5 Quantitative analysis limitations

FTIR can be used quantitatively, but it is not always simple. The main limitation for the quantitative purpose of FTIR is its accuracy, which can be influenced by sample heterogeneity, pathlength through the sample, and overlapping absorption bands. However, this requires the availability of accurate and reliable calibration curves, which can be problematic in some cases (*e.g.*, highly complex mixtures or low concentrations of target compounds).^172^

#### 5.2.6 Requirement for reference spectra

FTIR identification of materials is obscure if reference spectra are not available for comparison. Although there are large spectral libraries for many organic compounds, information on the reference spectra of inorganic materials is often more scarce or proprietary. This complicates efforts to analyze unknown compounds or materials that have not been previously well characterized.^176,178^

#### 5.2.7 Surface sensitivity and depth of penetration

FTIR tends to be more surface-sensitive, especially with techniques such as ATR. Which is sometimes an advantage and, at other times, a drawback, depending on the use case. This is an advantage for surface-sensitive studies, but it could give an incomplete view of the bulk composition. One drawback of this approach is that the infrared beam cannot penetrate too deeply into the samples, which limits its use in thick or multilayered samples. FTIR spectroscopy can be an invaluable method for inorganic material analysis, providing non-destructive, molecular–structure sensitivity and broad specificity. It also has its own limitations, such as difficulty in spectral interpretation, low sensitivity for specific materials, and limitations in quantitative analysis. Knowledge of these advantages and disadvantages is a prerequisite for the effective use of FTIR in material science as well as for reliable results.^78,103,147^

## 6 Comparison of FTIR with other analytical techniques: Raman and XRD in the nanoscale context

### 6.1 Spectral sensitivity to nanoscale features

Raman and FTIR spectroscopy are utilized to identify molecular structures and analyze chemical bonding in synthesized nanoparticles. Both methods reveal molecular vibrational modes, helping to determine specific functional groups and the surrounding chemical environment of NPs. Raman spectroscopy captures subtle vibrations of molecules with polarizability, while FTIR focuses on molecular bonds and functional groups possessing a permanent dipole moment. A drawback of Raman is the potential for fluorescence or sample damage caused by high-energy laser excitation, especially in sensitive biological materials. In contrast, FTIR uses longer wavelengths, which are generally less damaging, allowing multiple analyses without affecting sample integrity. Raman peaks often display weaker intensities compared to FTIR because they depend on changes in polarizability instead of direct absorption. Consequently, achieving similar peak strength may require extended acquisition times or higher sample concentrations. FTIR is particularly effective for studying bioorganic surface-associated components, whereas Raman demonstrates superior sensitivity to variations in allotropic forms and crystalline structures of NPs. XRD, on the other hand, is not vibrationally sensitive but detects long-range crystalline order—providing direct structural information such as unit cell dimensions, lattice strain, and crystallite size. Yet, for amorphous or poorly crystalline nanomaterials, XRD may provide limited data, whereas FTIR still delivers insight into local bonding environments.^179,180^

### 6.2 Surface *vs.* bulk information

In nanoscale systems, surface sensitivity is crucial. FTIR, particularly in ATR mode, probes just a few microns into the surface, making it ideal for studying surface coatings, functionalization, or degradation.^181–183^ Raman spectroscopy can also be surface-sensitive but typically requires optical transparency and is limited by fluorescence interference.^184^

XRD, by contrast, primarily provides bulk structural information, which may not reflect surface modifications or heterogeneity in nanoscale materials.^185^

### 6.3 Complexity and overlap in spectral interpretation

FTIR spectra of nanomaterials can exhibit overlapping vibrational bands, especially in complex mixtures (*e.g.*, MOFs with guest molecules, doped oxides, or mixed anion lattices). This makes deconvolution and interpretation challenging.^186–188^ Raman offers narrower peaks in many systems, enabling better resolution in complex structures.^189^

However, Raman is also prone to fluorescence interference, especially with organic linkers or dopants—where FTIR becomes more reliable.^184,190^

### 6.4 Quantitative detection of trace species

FTIR is typically semi-quantitative and struggles to detect trace-level species in nanocomposites, especially when matrix signals dominate.^191,192^ Raman can also suffer from low signal intensities for dilute species.^193^

In contrast, XRD can sometimes detect minor crystalline phases with high selectivity, but only if they are sufficiently crystalline and well-oriented.^194^ Advanced FTIR methods, such as TGA-FTIR coupling or signal-enhanced ATR-FTIR, are increasingly used to improve detection of minor components in nanoscale systems.^195,196^

### 6.5 *Operando* and *in situ* capability

All three techniques have *in situ* or *operando* variants, but FTIR is particularly well-suited for monitoring dynamic surface interactions, such as gas adsorption on nanocatalysts or ligand exchange in nanoparticles, especially under varying temperature, pressure, or humidity.^197,198^

Raman can provide similar capabilities, though sample heating and laser damage can be limitations.^199^ XRD-based *in situ* analysis is excellent for observing crystallographic phase transitions but less informative about surface chemistry.^200–202^

## 7 Future trends in FTIR spectroscopy for inorganic materials

The evolution of technology has driven the development of newer, more sophisticated instrument-based Fourier Transform Infrared spectroscopy (FTIR) capable of providing completely new pieces of evidence for in-depth investigations that broaden its application window, including those involving inorganic materials. In this section, we will analyze the technological advancements that have recently emerged along with FTIR and how they are defining new applications and research directions.^6,203^

### 7.1 Miniaturization and FTIR devices in a portable way

Improvement of miniaturization and portability of FTIR Spectrometers is one of the major trends seen in FTIR technology. These small instruments retain the features of a conventional colorimetric spectrometer used in laboratories but provide the convenience of off-site analysis. This is a breakthrough that will find significant applications in rapid, on-site measuring with a demand in fields such as environmental monitoring, archaeology, and industrial quality control. Portable FTIR has also found new applications in geological field work, allowing for immediate analysis of mineral compositions where accessibility is limited.^173,204^

### 7.2 Improved sensitivity and resolution

Recent advances in detector technology and optical components have resulted in FTIR spectrometers with improved sensitivity and resolution. These will enable the detection of low-level materials and close spectral overlapping features. Due to this, high-resolution FTIR instruments are now increasingly available in research areas that need accurate molecular characterization. This has particular relevance for in-depth investigations of complex inorganic materials such as mixed oxide phases or subtle structural differences.^205–207^

### 7.3 FTIR imaging and micro spectroscopy

Combining spatial resolution with spectral analysis, FTIR imaging, and microspectroscopy enables detailed mapping of the chemical composition of the surface of a sample. Modern data analysis, machine learning, graphical representation, and analytical tools, which build on classical techniques, will be important for data analysis in scanning X-ray imaging, especially for heterogeneous inorganic materials (*e.g.*, composites, ceramics, and mineral analysis with interest in the distribution of different components or different phases). Recent improvements in this field include accelerated data acquisition and advanced image processing algorithms, which allow for more precise and detailed chemical maps. Similarly, FTIR imaging is applicable to the investigation of corrosion processes or contaminant distribution in environmental samples.^173^

### 7.4 Integration with other analytical techniques

There is also an increasing trend in the use of FTIR in combination with other techniques such as Raman spectroscopy, X-ray diffraction (XRD), and scanning electron microscopy (SEM). Such a multi-modal approach facilitates a deeper analysis of all facets of inorganic materials by leveraging the advantages available through each technique. This way, FTIR in combination with other analytical techniques can provide complementary information (*e.g.*, vibrational information from Raman spectroscopy and other crystal structure data from XRD). These integrated systems are more prevalent than individual system components in advanced research labs, which have begun to employ them for a comprehensive view of material properties.^107,108,203^

### 7.5 Development of advanced ATR accessories

ATR (attenuated total reflectance) Attachment: ATR is one of the most common attachment apparatuses in FTIR spectroscopy, and it is particularly well suited for solid and liquid sample analysis. Newer developments in ATR technology are based on diamond-ATR crystals, which present the possibility for more rugged samples and a wider spectral range. Furthermore, freshly designed ATR types are coming on the market to get around challenging samples: highly absorbing materials, thin films. This enhanced approach to ATR-FTIR enables its application to many more inorganic materials while simplifying measurements, particularly for instrument-heavy use.^178,208^

### 7.6 Automation and artificial intelligence (AI) in FTIR

Employment of automation and artificial intelligence in FTIR spectroscopy—Automation, especially in terms of data processing and interpretation, is just starting to find a role. Automation of FTIR provides a number of benefits that facilitate the analysis, ranging from sample preparation to data acquisition and spectral analysis. For instance, AI algorithms are being designed to help interpret complex spectra by seeking patterns and predicting material properties *a priori* from spectral data. These innovations are decreasing analysis time while simultaneously increasing the accuracy and reproducibility of FTIR measurements.^209,210^

## 8 Emerging applications and research directions

### 8.1 Nanomaterials and thin films

With advancements in nanotechnology and increasing interest in the characterization of nanomaterials and thin films using FTIR. The ability of FTIR to probe surface chemistry and molecular interactions makes it a powerful tool for characterizing nanostructures. FTIR is used by researchers to understand the chemical composition and bonding environments of nanomaterials, including metal oxides, carbon-based materials, and thin semiconductor films. The development of dedicated accessories (*e.g.*, grazing angle ATR) extends the applications of FTIR to ultrathin films and ultra-thin surface layers.^10,205,211^

### 8.2 *In situ* and *operando* studies

For a meaningful interpretation of the structure and functionality of the material, *in situ* and *operando* FTIR studies are needed that can provide chemical complementary information within their actual dynamic states. This work consists of studying the materials in *operando* conditions while they experience chemical reactions, phase transformations, and/or other processes. *In situ* FTIR, for instance, is used to study the growth of corrosion products on metals, catalysts' oxidation, or activated cements' hydration. This method offers the most insight into reaction mechanisms and material behavior, which is crucial for designing new materials and optimizing current ones.^26,108^

### 8.3 Applications that relate to environmental and forensic sciences

Because of the demand for rapid and non-destructive analysis of complex samples, FTIR is seeing growth in environmental science applications as well as forensics. FTIR is used to determine the pollutants from water, soil, and air industries, along with microplastics analysis of environmental contaminants. FTIR is used in forensic science to analyze trace evidence such as fibres, paints, and explosives. Thus, the development of portable FTIR devices is crucial in these fields, as they can perform on-site analysis and help in making rapid decision-making.^91,212–214^

### 8.4 Cultural heritage and archaeology

FTIR is commonly used in cultural heritage analysis and conservation work, such as for artworks and historical or archaeological artifacts. Because FTIR is a non-destructive technique, it is perfect for analyzing delicate and priceless items. FTIR has been applied to pigment identification, binder and degradation product studies on artworks, and the composition of ancient ceramics, glass, and metal artifacts.^9^ Recent advances in FTIR imaging and micro spectroscopy are allowing for more detailed studies of these materials, resolving methods for understanding past manufacturing successes as well as cultural heritage aging processes.^6,166,173^

### 8.5 Energy materials and catalysis

The applications of FTIR in the emerging fields of energy materials and catalysis FTIR in terms of application. There are several fields where researchers have been applying FTIR to analyze chemical compositions and surface interactions in energy storage devices like batteries, fuel cells, and catalytic processes. *Operando* FTIR studies are highly valuable in this domain, as they enable the real-time monitoring of reactions and identification of active sites on the catalytic surfaces. Such knowledge is crucial to the design of more efficient energy conversion and storage technologies.^77,100,106,215^

### 8.6 Advanced materials characterization

Just as materials science is progressing, advanced characterization techniques with more information about complex materials are highly sought after. FTIR is used to study a wide range of advanced materials, including metal–organic frameworks (MOFs), perovskites, and hybrid organic-inorganic materials. FTIR researchers are finding new applications of FTIR along with an understanding of the molecular structure, bonding environments, and ways in which these materials interact with each other, leading to next-generation materials with engineered properties.^216–218^ FTIR spectroscopy has a promising future in the analysis of inorganic materials, with continuous improvements in technology and the widening of possible applications in the field. Advancements in miniaturization, ultrahigh sensitivity, FTIR imaging techniques, and combination with other analytical/pathological methods are broadening the scope of FTIR. Novel research directions are being motivated by emerging applications in nanomaterials, *in situ* studies, environmental science, and cultural heritage. With these trends continuing, FTIR will surely continue to be an important part of the chemist's toolbox, leading to the understanding of more complex inorganic materials as well as their properties.^205,216^

### 8.7 Biomedical and clinical diagnostics

Fourier Transform Infrared (FTIR) spectroscopy has emerged as a key analytical technique in biomedical and clinical diagnostics due to its ability to probe molecular vibrations without the need for dyes or labels. It has long been employed in qualitative assessments in the pharmaceutical and medical fields.^219^

More recently, FTIR has advanced to monitor biochemical changes at the cellular and subcellular levels, which is crucial for studying disease progression, cellular differentiation, and metabolic changes. The technique enables researchers to distinguish among healthy, apoptotic, and necrotic cells based on spectral patterns in the amide, lipid, and phosphate region.^220^ FTIR imaging and microspectroscopy have shown tremendous promise in medical histopathology, where the spatial resolution and chemical specificity of the technique allow differentiation of cancerous tissues from normal ones based on molecular fingerprinting. For example, amide I and II bands are sensitive to protein secondary structure, enabling the classification of tissue types without staining.^208^ Enzymatic assays can also be performed with FTIR when integrated with flow-through or microfluidic platforms. With appropriate calibration, FTIR allows real-time monitoring of enzymatic activity, offering new tools for drug discovery and clinical screening.^221^ Additionally, FTIR spectroscopy has been successfully used for real-time bioprocess monitoring, such as during fermentation or cell culture, by detecting key metabolites, proteins, and structural changes *in situ*. This allows researchers to optimize yields and detect deviations from normal physiological conditions promptly.^222^

### 8.8 Polymer science and smart materials

Fourier Transform Infrared (FTIR) spectroscopy is widely used to elucidate the molecular structure and chemical environment in polymers, including homopolymers, copolymers, and composites. By analyzing vibrational modes such as C–H, C=O, and C–O–C stretching, researchers can determine polymer composition, chain configuration, crystallinity, and crosslinking degree.^223^ Recent advancements in smart materials—such as shape-memory polymers, thermo-responsive hydrogels, and self-healing materials—have found FTIR essential for real-time monitoring of molecular reorganization under external stimuli like heat, light, or pH changes. For example, in temperature-sensitive poly(*N*-isopropylacrylamide) hydrogels, the collapse transition is observed through shifts in N–H and C=O stretching bands.^224^ In polymer–inorganic hybrids, FTIR detects the nature of bonding at the interface—such as hydrogen bonding or silane coupling—between organic chains and inorganic fillers like silica or clays. Spectral shifts in Si–O, B–O, or P–O bonds help determine the degree of chemical interaction and dispersion uniformity.^146,208^ ATR-FTIR (Attenuated Total Reflectance), due to its minimal sample preparation and surface sensitivity, is a preferred method for analyzing polymeric films and thin coatings. It provides a quick assessment of compositional changes in surface-treated or multi-layered systems.^225^ In FTIR microspectroscopy, spatial resolution allows mapping of chemical heterogeneity across a sample, revealing layer interfaces or defects in micro-structured materials like drug-release membranes and electronic packaging.^226^ Moreover, machine learning (ML) models are now being integrated with FTIR data to automate polymer classification, detect spectral anomalies, and predict physical properties. AI-driven spectral deconvolution offers higher consistency and interpretation accuracy, even in the presence of overlapping bands.^219^

## 9 Conclusion

Fourier Transform Infrared (FTIR) spectroscopy remains a vital tool for characterizing inorganic nanomaterials due to its ability to probe molecular vibrations, surface functionalities, and chemical interactions. However, its traditional applications often fall short of resolving the complexity introduced at the nanoscale, where factors such as particle size, surface functionalization, and quantum confinement effects play critical roles.

In recent years, the emergence of advanced FTIR methods—such as nano-FTIR, AFM-IR, and TGA-FTIR—has enabled researchers to overcome limitations like low spatial resolution and overlapping vibrational bands. These approaches offer enhanced surface sensitivity and allow real-time tracking of dynamic processes like gas adsorption, ligand exchange, and catalytic reactions in nanostructured systems. Despite these advancements, challenges remain in signal quantification for trace components, distinguishing surface from bulk contributions, and building spectral databases tailored to nanoscale environments.

To advance the field, future research should focus on integrating FTIR with complementary nanoscale characterization methods (*e.g.*, tip-enhanced Raman, XPS, or *in situ* XRD) and developing machine learning tools for deconvoluting complex spectra. Additionally, *operando* FTIR setups that mimic real environmental or catalytic conditions will be crucial for understanding functional behavior at the nanoscale. By refining FTIR methodologies and aligning them with the specific demands of nanoscience, this technique can continue to play a transformative role in materials research and nanotechnology.

## Author contributions

Kazi Al-Amin and Md. Kawsar collected the data and wrote the draft and original manuscript. Md. Tariqur Rahaman Bhuiyan Mamun assisted in collecting data and writing the manuscript. Md. Sahadat Hossain conceived and designed the review and analyzed the data.

## Conflicts of interest

The authors declare that they have no known competing financial interests or personal relationships that could have appeared to influence the work reported in this paper.

## Data Availability

No primary research results, software or code have been included and no new data were generated or analysed as part of this review.
